# Antioxidant Properties of Cap and Stipe from *Coprinus comatus*

**DOI:** 10.3390/molecules15031473

**Published:** 2010-03-09

**Authors:** Bo Li, Fei Lu, Xiaomin Suo, Haijuan Nan, Bin Li

**Affiliations:** 1 School of Food Science, Henan Institute of Science and Technology, Xinxiang 453003, Henan, China; E-Mails: lufei709@yahoo.com.cn (F.L.); nanhaijuan1@163.com (H.N.); 2 School of Food Science, Huazhong Agricultural University, Wuhan 430070, Hubei, China; E-Mails: suoxiaominbaby@126.com (X.S.); libinfood@yahoo.com.cn (B.L.)

**Keywords:** *Coprinus comatus*, cap, stipe, antioxidant property

## Abstract

*Coprinus comatus*, also called chicken drumstick mushroom, is currently commercially available in China. Hot water and ethanolic extracts were prepared from cap and stipe of *C. comatus* fruit bodies and their antioxidant properties were studied. Ethanolic extract from stipe showed high antioxidant activity (80.6%) at 1 mg/mL. Reducing power of hot water extracts from cap was 1.653 at 10 mg/mL. Extracts from cap showed better scavenging ability on DPPH (57.9% at 1 mg/mL) than stipe ones. Ethanolic extracts were more effective in scavenging ability on hydroxyl radicals (57.4–61.3% at 5 mg/mL) than hot water extracts. Ethanolic extracts showed moderate scavenging ability on superoxide radicals (46.3–47.0% at 20 mg/mL). Naturally occurring antioxidant components including total phenols (3.60–20.00 mg/g), tocopherols (0.58–11.93 mg/g), flavonoids (0.19–3.52 mg/g) and polysaccharides (58.52–547.86 mg/g) were found in the extracts. Overall, extracts from cap were more effective for the antioxidant properties assayed.

## 1. Introduction

*Coprinus comatus* (Müll.) Gray, also known as chicken drumstick mushroom, the shaggy ink cap, lawyer’s wig or shaggy mane, is a novel cultivated edible mushroom in China. For its good nutritional properties, delicious taste and particular figure (like chicken drumstick), more and more people like to eat the mushroom, and its output in China in 2006 was 382,000 tons. Various bioactive functions of *C. comatus* have been reported in recent years, such as hypoglycemic, immunomodulation, hypolipidemic, antitumor and antibacterial effects [1,2]. 

Many studies have shown that natural antioxidants in plants are closely related to their bioactivities such as the reduction of chronic diseases and inhibition of pathogenic bacteria growth, which are often associated with the termination of free radical propagation in biological systems. Fruit bodies and mycelia of several mushrooms have been reported to show the antioxidant activities [3,4,5]. Over fifty years ago, *C. comatus* was found to contain ergothioneine, a thiol compound with antioxidant properties [6]. The antioxidant activity was later confirmed by Badalyan *et al.* in 2003 [7]. Antioxidant properties of *C. comatus* fruit bodies, mycelia and fermentation filtrate were reported by Tsai *et al.* [8]. The selenium-polysaccharide isolated from selenium-enriched mycelia of *C. comatus* caused a significant decrease in the level of malondialdehyde (MDA) and a significant increase in the activities of enzymic antioxidants and the levels of non-enzymic antioxidants in liver and kidney of diabetic mice [9]. However, the differences of antioxidant properties between the cap and stipe of *C. comatus* have not been reported till now.

The cap and stipe of *C. comatus* are easily separated. Because the cap will autodigest quickly after collection, separating the cap and stipe benefits greatly the processing and utilization of *C. comatus*. Our previous study found that there were some differences on chemical components between cap and stipe of *C. comatus* [10]. Therefore, the objective of this work was to evaluate any differences in antioxidant properties of ethanolic and hot water extracts of cap and stipe of *C. comatus*, including antioxidant activity, reducing power and scavenging abilities on radicals. The contents of potential antioxidant components in these extracts were also determined.

## 2. Results and Discussion 

### 2.1. Extraction Yield

The extraction yields of hot water extracts from stipe and cap were higher than those of ethanolic extracts. Furthermore, both hot water and ethanolic extracts from cap were higher than those from stipe (Table 1). The difference in the yields of hot water and ethanolic extracts might be due to the fact that *C. comatus* contained more water-soluble components such as soluble polysaccharides than ethanolic-soluble components.

### 2.2. Antioxidant Activity

**Figure 1:**
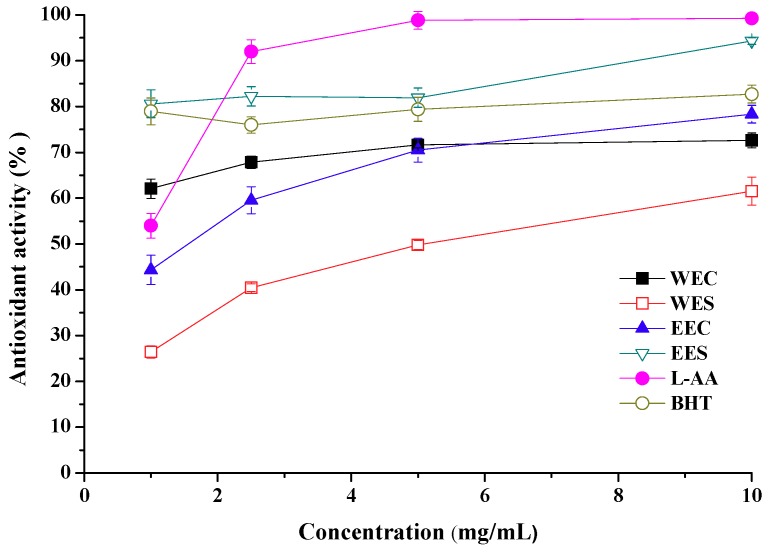
Antioxidant activity of ethanolic and hot water extracts from stipe and cap of *Coprinus comatus*.

The antioxidant activity assayed was the ability to inhibit the peroxidation of linoleic acid. Using the ferric thiocyanate method, the antioxidant activity was determined after incubation at 37 °C for 24 h. Antioxidant activities of ethanolic extracts from stipe and cap were 80.6% at 1 mg/mL and 70.5% at 5 mg/mL, respectively (Figure 1). For hot water extracts, antioxidant activities from stipe and cap were 61.5% and 72.6% at 10 mg/mL, respectively. Furthermore, antioxidant activities of extracts increased with concentration enhancement from 1 to 10 mg/mL. Generally, for both extracts, antioxidant activities were in the descending order: ethanolic extract from stipe (EES) >ethonalic extract from cap (EEC) ≈ hot water extract from cap (WEC) >hot water extract from stipe (WES). Moreover, antioxidant activity of EES was higher than L-ascorbic acid (L-AA, 54.0%) and butylated hydroxytoluene (BHT, 78.9%) at 1 mg/mL, and was close to L-AA (99.3%) and higher than BHT (82.7%) at 10 mg/mL. It can be seen that ethanolic extract from stipe of *C. comatus* showed distinctive antioxidant activity comparing with other mushrooms [3,4,5].

**Figure 2:**
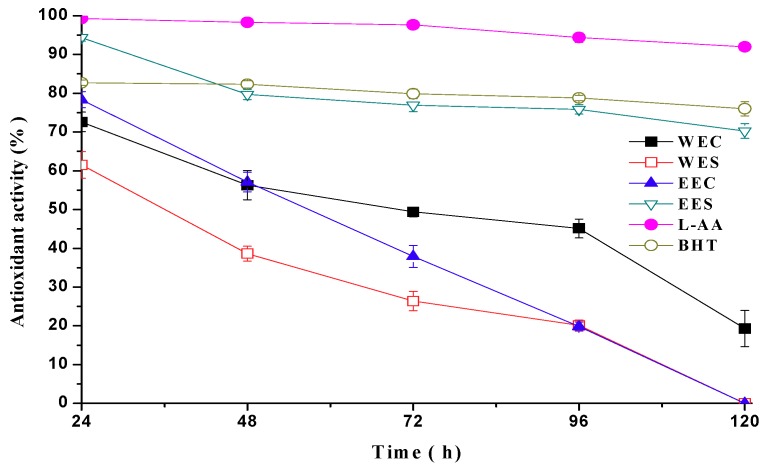
Change of antioxidant activity of various extracts from *Coprinus comatus* with time prolongation.

To determine the stability of antioxidant in the linoleic acid emulsion, antioxidant activity of various extracts from *C. comatus* were measured at 10 mg/mL per 24 h five times (Figure 2). Antioxidant activities of WEC, WES and EEC decreased distinctly with time prolongation. WES and EEC didn’t show antioxidant activity after 120 h. However, antioxidant activity of EES was reasonably stable in the linoleic acid system and there was only slight decline on antioxidant activity after 120 h. Therefore, EES possessed a high and stable antioxidant activity.

### 2.3. Reducing Power

**Figure 3:**
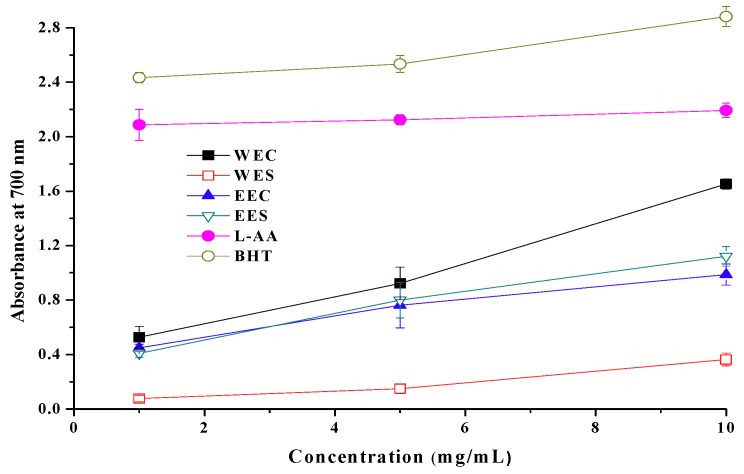
Reducing power of ethanolic and hot water extracts from stipe and cap of *Coprinus comatus*.

Hot water extracts from cap and stipe showed reducing powers of 1.653 and 0.364 at 10 mg/mL, respectively (Figure 3). For ethanolic extracts of cap and stipe, reducing powers were 0.998 and 1.122 at 10 mg/mL, respectively. Apparently, hot water extract from cap showed highest reducing power among four extracts. Generally, for both extracts, reducing powers in the descending order: WEC > EES > EEC > WES. However, BHT and L-ascorbic acid showed excellent reducing powers of 2.834 and 2.087 at 1.0 mg/mL.

The antioxidant activity has been reported to be concomitant with the development of reducing power [11]. Therefore, the antioxidant activity of the extracts might partially be a result of its reducing power. However, WEC showed the highest reducing power among four extracts, which was different from antioxidant activity experiment where EES showed the best activity. It can be indicated that chemical components affecting the reducing power and antioxidant activity were different to some extent.

### 2.4. Scavenging Ability on 1,1-Diphenyl-2-picrylhydrazyl (DPPH) Radicals

**Figure 4:**
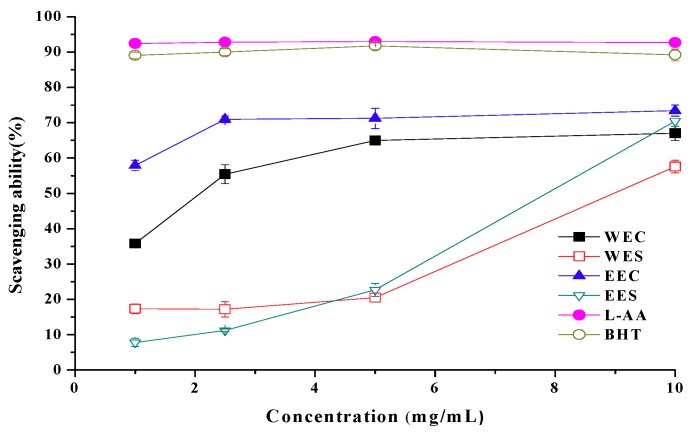
Scavenging ability of ethanolic and hot water extracts from stipe and cap of *Coprinus comatus* on DPPH radicals.

Ethanolic and hot water extracts from cap showed scavenging abilities of 57.9% and 35.8% at 1 mg/mL, whereas scavenging abilities of ethanolic and hot water extracts from stipe were 22.7% and 20.5%, respectively (Figure 4). Apparently, extracts from cap showed better scavenging ability on DPPH than extracts from stipe. However, at 1 mg/mL, BHT and L-ascorbic acid showed high scavenging abilities of 89.0% and 92.4%, respectively.

DPPH radical is a stable free radical, which can accept an electron or hydrogen radical to become a stable diamagnetic molecule. The reduction capability of DPPH radical was determined by the decrease in its absorbance at 517 nm induced by antioxidants. This chromogen radical compound can directly react with antioxidants and the procedure is simple, rapid and sensitive. Hence, the model of scavenging the stable DPPH radical is a widely used method to evaluate the free radical scavenging ability of various samples [12]. Experimental results showed that ethanolic and hot water extracts from *C. comatus*, especially from cap, contained antioxidant components, which could react rapidly with DPPH radicals.

### 2.5. Scavenging Ability on Hydroxyl Radicals

**Figure 5:**
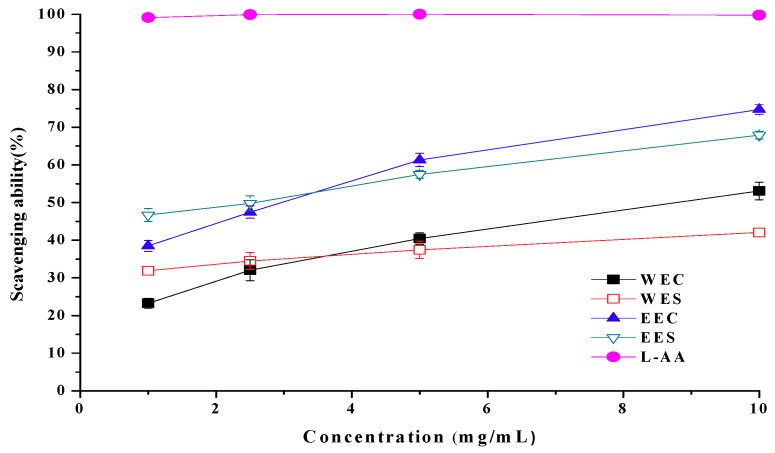
Scavenging ability of ethanolic and hot water extracts from stipe and cap of *Coprinus comatus* on hydroxyl radicals.

Hydroxyl radicals are highly reactive and therefore potentially damaging to cells, in particular by initiating lipid peroxidation. The method of Smirnoff is convenient for the selection of hydroxyl radical scavenger. Ethanolic extracts from cap and stipe showed scavenging abilities on hydroxyl radicals of 61.3% and 57.4% at 5 mg/mL, whereas scavenging abilities of hot water extracts were 40.5% (cap) and 35.4% (stipe) at 5 mg/mL, respectively (Figure 5). Apparently, ethanolic extracts from *C. comatus* were more effective in scavenging ability on hydroxyl radicals than hot water extracts. However, the scavenging ability of L-ascorbic acid was almost 100% at any selected concentration.

### 2.6. Scavenging Ability on Superoxide Radicals

**Figure 6:**
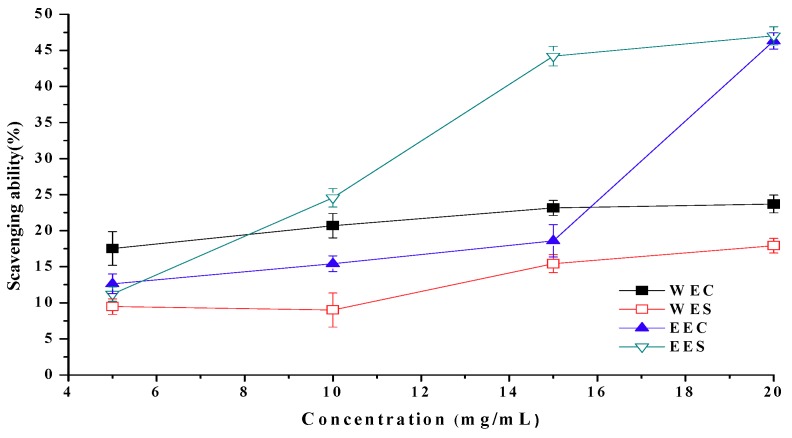
Scavenging ability of ethanolic and hot water extracts from stipe and cap of *Coprinus comatus* on superoxide radicals.

Superoxide ion is the one-electron reduction product of oxygen. While superoxide radical can be directly toxic, it has a limited reactivity with lipids, raising questions about its true toxicity [13]. Ethanolic extracts from cap and stipe showed scavenging abilities on superoxide radicals of 47.0% and 46.3% at 20 mg/mL, respectively (Figure 6). Scavenging abilities of hot water extracts were 23.7% and 17.9% at 20 mg/mL, respectively. Generally, for both extracts, scavenging ability on superoxide radicals in the descending order: EES > EEC > WEC > WES. However, the scavenging ability of L-ascorbic acid was up to 90% at 1 mg/mL (not shown in Figure 6).

### 2.7. EC_50_ Values in Antioxidant Properties

The antioxidant properties assayed herein were summarized in Table 2 and the results were normalized and expressed as EC_50_ values (mg various extracts per mL) for comparison. Effectiveness of antioxidant properties inversely correlated with their EC_50_ values. Generally, ethanolic extracts were more effective than hot water extracts in antioxidant activity, scavenging abilities on DPPH radicals, hydroxyl radicals and superoxide radicals, whereas hot water extract from cap was more effective in reducing power. With regard to ethanolic extracts, extracts from cap were more effective than extracts from stipe in reducing power, scavenging abilities on DPPH radicals and hydroxyl radicals, whereas the latter were more effective in antioxidant activity and scavenging ability on superoxide radicals. For hot water extracts, extracts from cap were more effective than extracts from stipe in all antioxidant properties.

### 2.8. Antioxidant Components

Several potential antioxidant components including total phenols, flavonoids, tocopherols, ascorbic acid and polysaccharide were determined (Table 3).

Phenols are known to be effective antioxidants in plants due to their hydroxyl groups [14]. Typical phenols with antioxidant activity have been characterized as phenolic acids and flavonoids. Phenolic acids have repeatedly been implicated as natural antioxidants in fruits, vegetables and other plants [15,16]. Total phenols were found in four extracts of *C. comatus* (3.60–20.00 mg/g). Extracts from cap contained more total phenols than extracts from stipe, especially hot water extract of cap containing the maximum phenols. It might explain that ethanolic extract from cap was more effective than that from stipe in reducing power, scavenging abilities on DPPH radicals and hydroxyl radicals, hot water extract from cap was more effective than that from stipe in all antioxidant properties, and hot water from cap showed the highest reducing power among four extracts.

Flavonoids act as antioxidant agents by direct free radical scavenging, transition metal chelation and maintenance of endogenous antioxidants such as the glutathione and superoxide dismutase systems [17]. Total flavonoids were detected in four extracts of *C. comatus* (0.19–3.52 mg/g). Furthermore, ethanolic extracts contained more flavonoids than hot water extracts. Tocopherols were found mainly in ethanolic extracts of cap and stipe, which might be responsible for their higher antioxidant properties than hot water extracts. Moreover, the content of tocopherols in ethanolic extracts from *C. comatus* was 11.74–11.93 mg/g, which was quite high among reported mushrooms. Accordingly, extracts of *C. comatus* possess effective antioxidant activities. Ascorbic acid was not detected in four extracts, which might be due to that ascorbic acid was easily oxidized in hot water and difficult to resolve in ethanol. Polysaccharides extracted from several mushrooms have shown antioxidant properties for their free radical scavenging ability [18,19]. Hot water extracts of *C. comatus* contained a considerable quantity of polysaccharides (269.73–547.86 mg/g) and their content in stipe was higher than in cap. These polysaccharides might contribute in part to the antioxidant properties of hot water extracts to compensate their lack in tocopherols to some degree.

## 3. Experimental

### 3.1. Materials

Fresh fruit bodies of *C. comatus* were obtained from the Xinxiang City, Henan Province, China. The stipe and cap of fruit bodies were peeled off and cut half, then air-dried in an oven (beginning at 30 °C, then increasing 5 °C every 3 h until 45 °C), respectively. The dried cap and stipe were milled into powder (40 mesh). For each of ethanolic and hot water extractions from stipe and cap, three dried samples (~50 g each) were randomly selected and prepared for analyses. BHT, ascorbic acid, DPPH, flavonoid and tocopherol were purchased from Sigma. The other chemicals and reagents used were of analytical grade. 

### 3.2. Extraction

For ethanolic extraction, a subsample (20 g) was extracted by stirring with ethanol (200 mL, 95% pure) at 25 °C for 24 h and filtering through Whatman No.1 filter paper. The residue was then extracted with two additional 200 mL portions of ethanol as described above. The combined ethanolic extracts were then rotary evaporated at 40 °C to dryness.

For hot water extractions, a subsample (20 g) was heated with deionized water (200 mL) at reflux for 1 h, centrifuging at 4,000 rpm for 15 min and filtering through Whatman No.1 filter paper. The residue was then extracted with two additional 200 mL portions of boiling water as described above. The combined hot water extracts were freeze-dried, respectively. The dried extract was used directly for analyses of antioxidant components or redissolved in water or ethanol to a concentration of 50 mg/mL and stored at 4 °C for further uses.

### 3.3. Antioxidant Activity

The antioxidant activity was carried out by using a linoleic acid system [20]. The linoleic acid emulsion was prepared by mixing linoleic acid (0.28 g), Tween 20 (0.28 g) as emulsifier, and 0.2 M phosphate buffer (50 mL, pH 7.0). Each extract (1–10 mg/mL) in water or ethanol (0.5 mL) was mixed with 0.2 M linoleic acid emulsion (2.5 mL) and 0.2 M phosphate buffer (2 mL, pH 7.0). The reaction mixture was incubated at 37 °C in the dark to accelerate the peroxidation process. The levels of peroxidation were determined according to the thiocyanate method by sequentially adding 75% ethanol (5 mL), 30% ammonium thiocyanate (0.1 mL), sample solution (0.1 mL) and ferrous chloride in 3.5% HCl (0.1 mL). After the mixture was kept for 3 min, the peroxide value was measured at 500 nm against a blank in a Unico 7200 spectrophotometer (Unico, China). The antioxidant activity was calculated as follows: antioxidant activity (%) = [(△A_500_ of control - △A_500_ of sample)/△A_500_ of control] × 100. A value of 100% indicates the strongest antioxidant activity. EC_50_ value (mg extract/mL) is the effective concentration at which the antioxidant activity was 50% and was obtained by interpolation from linear regression analysis. Furthermore, the antioxidant activity was determined per every 24 h. Ascorbic acid and BHT were used for comparison.

### 3.4. Reducing Power

The reducing power was determined according to the method of Oyaizu [21], which measured the power of sample to reduce ferricyanide to ferrocyanide. Each extract (1–10 mg/mL) in water or ethanol (2.5 mL) was mixed with 0.2 M phosphate buffer (2.5 mL, pH 6.6) and 10 mg/mL potassium ferricyanide (2.5 mL), and the mixture was incubated at 50 °C for 20 min. After trichloroacetic acid (2.5 mL, 100 mg/mL) was added, the mixture was centrifuged at 3,000 rpm for 10 min. The upper layer (5 mL) was mixed with deionized water (5 mL) and 1 mg/mL ferric chloride (1 mL), and the absorbance was measured at 700 nm against a blank. A higher absorbance indicates a higher reducing power. EC_50_ value (mg extract/mL) is the effective concentration at which the absorbance was 0.5 for reducing power and was obtained by interpolation from linear regression analysis. Ascorbic acid and BHT were used for comparison.

### 3.5. Scavenging Ability on DPPH Radicals

The scavenging ability on DPPH radicals was determined according to the method of Shimada *et al.* [22]. Each extract (1–10 mg/mL) in water or ethanol (2 mL) was mixed with methanolic solution (1 mL) containing DPPH radicals, resulting in a final concentration of 0.2 mM DPPH. The mixture was shaken vigorously and left to stand for 30 min in the dark, and the absorbance was then measured at 517 nm against a blank. EC_50_ value (mg extract/mL) is the effective concentration at which DPPH radicals were scavenged by 50% and was obtained by interpolation from linear regression analysis. Ascorbic acid and BHT were used for comparison.

### 3.6. Scavenging Ability on Hydroxyl Radicals

Scavenging ability on hydroxyl radicals was determined according to the method of Smirnoff *et al.* [23] with some modifications. Hydroxyl radicals from the Fenton reaction can react with salicylate to form a colored material (2,3-dihydroxybenzoate). Scavenging activity of hydroxyl radical can be assayed by the color change of reaction system. Each extract (1–10 mg/mL) in water or ethanol (1 mL) was mixed with 9 mmol/L FeSO_4_ (1 mL) and 9 mmol/L salicylic acid in 95% ethanol (1 mL). The reaction was initiated by the addition of 8.8 mmol/mL H_2_O_2_ (1 mL). After 0.5 h at 37 °C, the absorbance of the mixture was determined at 510 nm against a blank. Mixture without sample was the control and mixture without H_2_O_2_ was the blank. The scavenging activity was calculated as follows: scavenging activity (%) = [1-(△A_510_ of sample –△A_510_ of blank)/△A_510_ of control] × 100. EC_50_ value (mg extract/mL) was calculated according to the relationship of concentration and scavenging activity. Ascorbic acid was used for comparison.

### 3.7. Scavenging Ability on Superoxide Radicals

Pyrogallol (1,2,3-benzenetriol) can autoxidize rapidly, especially in alkaline solution, and produce superoxide anion. Scavenging ability on superoxide radicals was determined according to the method of Marklund [24] with some modifications described by Yu *et al* [25]. Each extract (5–20 mg/mL) in water or ethanol (0.1 mL) was mixed with 50 mmol/L tris-hydrochloric acid buffer (3 mL, pH 8.2). After 20 min at 25 °C, 7 mmol/L pyrogallol (3 mL) was added and the mixture was shaken rapidly. After 4 min, the reaction was terminated by the addition of 10 mol/L hydrochloric acid (0.5 mL) and the absorbance of mixture was determined at 420 nm. Mixture without sample was the control and mixture without pyrogallol was the blank. The scavenging activity was calculated as follows: scavenging activity (%) = [1-(△A_420_ of sample –△A_420_ of blank)/△A_420_ of control) ×100. EC_50_ value (mg extract/mL) was calculated according to the relationship of concentration and scavenging activity. Ascorbic acid was used for comparison.

### 3.8. Determination of Antioxidant Components

Total phenols was determined according to the method of Singleton *et al.* [26] with a slight modification. Each extract in water or ethanol (0.4 mL) was mixed with distilled water (9.6 mL) and Folin-Ciocalteu reagent (1 mL). After 5 min at 22 °C, 5% sodium carbonate (5 mL) was added. After 60 min of standing at 22 °C, absorbance was measured at 750 nm against a blank. The content of total phenols was calculated on the basis of the calibration curve of gallic acid.

The total flavonoid was determined according to the method of Jia *et al.* [27] with a slight modification. Each extract in water or ethanol (1 mL) was mixed with 30% ethanol (4 mL) and 5% NaNO_2_ (0.3 mL). After 6 min at 25 °C, 10% Al(NO_3_)_3_ (0.3 mL) was added. Then 4% NaOH (4 mL) and 30% ethanol (0.4 mL) was added after 6 min. The mixture was thoroughly mixed and kept for 12 min. Absorbance of the mixture was determined at 510 nm against a blank. The content of total flavonoid was calculated on the basis of the calibration curve of rutin.

Tocopherols were analyzed according to the Chinese GB/T 5413.9–1997 method. Each dried ethanol or water extract (1 g) was suspended in 50 °C distilled water (30 mL). Then 1.5% ascorbic acid solution (in ethanol, 100 mL) and 50% potassium hydroxide solution (50 mL) were added, and the mixture was saponified at 100 °C for 30 min. Water (100 mL) was added and the mixture was extracted with petroleum ether (100 mL). The organic layer was washed with distilled water until neutral, died over anhydrous sodium sulfate and rotary evaporated to dryness. The residue was dissolved in petroleum ether and transferred to volumetric flask. Petroleum ether was blown to dryness, and the residue was dissolved in methanol (5 mL) for HPLC analysis. The mobile phase was methanol, at a flow rate of 1.0 mL/min and UV detection was 294 nm. The HPLC instrument was Waters 2695 (Waters, USA) with an ODS C-18 column.

Ascorbic acid was assessed using the 2,6-dichloro-indophenol titration method [28]. Polysaccharide was determined according to the phenol-sulfuric acid method [29]. Test solution (1 mL) was mixed with distilled water (1 mL), 6% phenol (1 mL) and concentrated sulfuric acid (5 mL). After 10 min of standing, the mixture was shaken and kept for an additional 20 min. Absorbance was measured at 490 nm against a blank. The content of polysaccharide was calculated on the basis of the calibration curve of glucose.

### 3.9. Statistical Analysis

All the experimental results were the mean (±standard deviation) of three parallel measurements. The efficient concentration of antioxidant required to induce a 50% effect, the EC_50_ value, was obtained by interpolation from the linear regression analysis and was expressed as the concentration of extract (mg/mL). The lower the EC_50_ value is, the more efficient the sample is. Data were evaluated by using one-way variance analysis P values < 0.05 were regarded as significant and p < 0.01 as very significant.

## 4. Conclusions 

Ethanolic extracts of *C. comatus* were more effective than hot water extracts in antioxidant activity, scavenging abilities on DPPH radicals, hydroxyl radicals and superoxide radicals, whereas hot water extract from cap was more effective in reducing power. With regard to cap and stipe, ethanolic extracts from cap were more effective than that from stipe in reducing power, scavenging abilities on DPPH radicals and hydroxyl radicals, whereas the latter were more effective in antioxidant activity and scavenging ability on superoxide radicals. Furthermore, hot water extracts from cap were more effective than those from stipe in all antioxidant properties. Extracts from cap contained more total phenols than stipe, ethanolic extracts contained more flavonoids than hot water extracts, and tocopherols were found mainly in ethanolic extracts, which might explain the higher antioxidant properties in cap extracts and ethanolic extracts. The considerable quantity of polysaccharides in hot water extracts might contribute in part to the antioxidant properties. The results showed that extracts from *C. comatus* were effective in antioxidant properties. For the differences of chemical components, antioxidant activities and storable properties between the cap and stipe, separating the cap and stipe will be beneficial for the processing and utilization of this mushroom.

## Figures and Tables

Each value is expressed as mean ± S.D. (n = 3).

Each value is expressed as mean ± S.D. (n = 3).

Each value is expressed as mean ± S.D. (n = 3).

Each value is expressed as mean ± S.D. (n = 3).

Each value is expressed as mean ± S.D. (n = 3).

Each value is expressed as mean ± S.D. (n = 3).

**Table 1:** Extraction yield of various extracts from *C. comatus*.

Extract	Extraction yield ^a^ (g/100 g dry weight)	
	Ethanolic	Hot water
Stipe	11.7 ± 0.7	46.5 ± 2.3
Cap	14.5 ± 1.1	49.2 ± 0.6

^a^ Extracted from air-dried materials. Each value is expressed as mean ± S.E. (n = 3).

**Table 2:** EC_50_ values of ethanolic and hot water extracts from cap and stipe of *Coprinus comatus* in antioxidant properties.

Extract	Antioxidant attribute	EC_50_^a^ (mg/mL)	
		Cap	Stipe
Ethanolic	Antioxidant activity Reducing power Scavenging ability on DPPH radicals Scavenging ability on OH radicals Scavenging ability on superoxide radicals	1.56 ± 0.24 ^b^ 1.67 ± 0.13 0.86 ± 0.06 3.23 ± 0.28 25.3 ± 0.21	0.62 ± 0.42 1.93 ± 0.23 7.86 ± 0.16 3.35 ± 0.17 20.7 ± 0.38
Hot water	Antioxidant activity Reducing power Scavenging ability on DPPH radicals Scavenging ability on OH radicals Scavenging ability on superoxide radicals	0.81 ± 0.03 0.95 ± 0.05 2.09 ± 0.26 8.66 ± 0.35 -- ^c^	5.08 ± 0.17 14.8 ± 0.22 8.98 ± 0.19 16.9 ± 0.41 --

^a^ EC_50_ value: The effective concentration at which the antioxidant activity was 50%; the absorbance was 0.5 for reducing power; DPPH radicals, hydroxyl radicals or superoxide radicals was scavenged by 50%, respectively. EC_50_ value was obtained by interpolation from linear regression analysis. ^b^ Each value is expressed as mean ± S.D. (n = 3). ^c^ No effect.

**Table 3:** Contents of total phenols, total flavonoid, tocopherols, ascorbic acid and polysaccharide of various extracts from *Coprinus comatus*.

Antioxidant components		Content (mg/g)	
		Ethanolic	Hot water
Stipe	Total phenols	9.71 ± 0.26 ^a^	3.60 ± 0.64
	Total flavonoid	3.52 ± 0.20	0.19 ± 0.02
	α-Tocopherol	11.1 ± 0.14	0.52 ± 0.02
	γ-Tocopherol	0.20 ± 0.01	0.05 ± 0.01
	δ-Tocopherol	0.45 ± 0.03	0.01 ± 0.01
	Ascorbic acid	Nd ^b^	nd
	Polysaccharide	64.4 ± 2.56	547 ± 4.63
Cap	Total phenols	13.5 ± 1.18	20.0 ± 0.44
	Total flavonoid	2.13 ± 0.31	1.88 ± 0.39
	α-Tocopherol	11.0 ± 0.18	0.64 ± 0.03
	γ-Tocopherol	0.29 ± 0.01	0.04 ± 0.01
	δ-Tocopherol	0.62 ± 0.04	0.03 ± 0.01
	Ascorbic acid	nd	nd
	Polysaccharide	58.5 ± 3.12	269.7 ± 6.64

^a^ Each value is expressed as mean ± S.D. (n=3). ^b^ Not detected.
